# Large extracellular vesicles derived from LPS-preconditioned cardiomyocytes alleviate myocarditis via mediating macrophage polarization and modulating p38 MAPK pathway

**DOI:** 10.3389/fimmu.2025.1629676

**Published:** 2025-09-09

**Authors:** Yanjie Jiang, Yingnan You, Yaxue Xie, Shan Zhou, Mengjie Ma, Bo Han

**Affiliations:** ^1^ Department of Pediatric Cardiology, Shandong Provincial Hospital Affiliated to Shandong First Medical University, Jinan, Shandong, China; ^2^ Department of Pediatrics, The Affiliated Taian City Central Hospital of Qingdao University, Tai’an, Shandong, China; ^3^ School of Medicine, Cheeloo College of Medicine, Shandong University, Jinan, Shandong, China; ^4^ Shandong First Medical University & Shandong Academy of Medical Sciences, Jinan, Shandong, China

**Keywords:** myocarditis, cardiomyocytes, macrophages, large extracellular vesicles, MAPK

## Abstract

**Introduction:**

Myocarditis is an inflammatory injury to the myocardium characterized by disrupted intercellular communication, involving macrophages and cardiomyocytes as key players. However, the interactions between macrophages and cardiomyocytes during myocarditis remain inadequately explored. Emerging evidence indicated that extracellular vesicles (EVs) play a crucial role in intercellular communication.

**Methods:**

In our study, LPS- or PBS-preconditioned cardiomyocytes derived large EVs (C-lEV_LPS_/C-lEV_PBS_) were isolated. qPCR, ROS and flow cytometry assays were employed to evaluate their impact on macrophages and in the in vivo experiments, C-lEV_LPS_ was administered to mice with viral myocarditis. Cardiac function was assessed through echocardiography and cTnT levels, while inflammatory responses were analyzed via histopathological examination and cytokine profiling. Then mechanistic investigations were performed using integrated transcriptomic and proteomic profiling to characterize EV-mediated regulatory networks. Statistical analyses were performed using Student’s t-test or ANOVA, with significance set at *p* < 0.05.

**Results:**

We demonstrated C-lEV_LPS_ exhibited anti-inflammatory effects on macrophages and alleviated cardiac inflammation and dysfunction in a mouse model of CVB3-induced myocarditis. Additionally, C-lEV_LPS_ facilitated macrophage polarization toward the M2-like phenotype and inhibits M1 polarization, both in vitro and in vivo. Notably, compared to C-lEV_PBS_, C-lEV_LPS_ was enriched in the phosphatase 2 scaffold subunit alpha protein (PP2AA), which can recruit other subunits to form the PP2A complex, ultimately leading to the dephosphorylates of p38.

**Discussion:**

This study highlights the effect of C-lEV_LPS_ in myocarditis and uncovers the potential mechanism that modulates macrophage polarization by delivering PP2AA from cardiomyocytes to macrophages and regulating the p38 MAPK pathway. These findings provide a promising therapeutic strategy for myocarditis.

## 1 Introduction

Myocarditis, an inflammation of the heart muscle, presents a range of symptoms from mild fatigue and chest discomfort to severe complications such as arrhythmias, heart failure, cardiogenic shock, and sudden death (1, 2). The etiology of myocarditis is multifactorial, with common infectious agents including bacteria, viruses, fungi, and parasites. In developed countries, viral infections (such as Coxsackie virus B3 and adenovirus) are the most prevalent causes (1); whereas in tropical and subtropical regions, parasitic infections such as Chagas disease, toxoplasmosis, and schistosomiasis contribute significantly to the incidence of myocarditis (3). These pathogens can induce myocardial inflammation through direct invasion or immune-mediated mechanisms. Due to the incomplete understanding of myocarditis pathogenesis, specific treatment strategies are currently unavailable (1, 2).

Cardiomyocytes, the primary cellular components of the heart, are targeted during myocarditis by pathogens like viruses, leading to their replication within the host cells (1, 2, 4, 5). This results in direct damage characterized by swelling, apoptosis, and necrosis (4). Additionally, macrophages—the main immune cells in the heart (1, 5, 6)—accumulate at injury sites during myocarditis and exhibit significant phenotypic plasticity (6, 7). They are generally classified into M1 and M2 macrophages, where M1 macrophages enter injury sites and secrete pro-inflammatory cytokines that block tissue repair, while M2 macrophages aid in later healing stages by eliminating pathogens and encouraging the repair of inflammatory sites (8–10). Given the dynamic and heterogeneous nature of macrophages, targeting macrophage heterogeneity may offer new therapeutic avenues for myocarditis.

Communication between macrophages and cardiomyocytes usually occurs via direct contact, paracrine factors, or extracellular vesicles (EVs) (11). EVs are now recognized as critical modulators in both physiological and pathological processes, especially in contexts such as cardiovascular diseases (12–17). Notably, EVs serve as key mediators by transferring cellular substances like miRNA and proteins (18, 19). For instance, hypoxic cardiomyocytes release small EVs rich in TNF-α (20), while exosomes originating from M2 macrophages containing miRNA-148a and miRNA-378a-3p mitigate cardiomyocyte apoptosis and pyroptosis following cardiac injury (19, 21). In the context of heart failure and myocardial infarction, EVs play crucial roles in cardiac repair, especially when triggered by cell therapy (22–24).

According to the MISEV 2023 recommendations, EVs are classified into small EVs (sEVs) and large EVs (lEVs, also referred to as microvesicles) based on size (25). Although sEVs might generally refer to EVs <200 nm in diameter and lEVs typically ranging from 150 nm to over 1,000 nm, there is no strict consensus on upper and lower size cut-offs (25, 26). And it has become clear that many separation methods, such as dUC, yield EV subpopulations with overlapping size distributions (25). Critically, compared with sEVs, lEVs carry unique bioactive molecules, such as specific protein signatures (27–29) and potentially distinct sets of miRNAs (30). These cargo molecules often fulfilling roles that differ from those attributed to sEVs (29), contributing significantly to processes like immunomodulation (31, 32) and tissue repair (9), Meanwhile, their larger size confers greater cargo capacity, enabling them to transport complex molecular or larger volumes of bioactive cargo for enhanced functional effects upon fusion with recipient cells (33) and larger membrane area enhancing their fusion ability with recipient cells (34). However, in myocarditis—an immune-mediated myocardial injury—the signaling functions and pathological regulatory mechanisms of EVs, particularly cardiomyocyte-derived lEVs (C-lEVs) with unique large cargo transport capacity, remain significantly understudied. Urgent multi-dimensional investigations are needed to uncover their potential value, thus offering new opportunities for the accurate diagnosis and targeted therapy of myocarditis.

This research investigated the impact of C-lEVs on myocarditis, focusing on the interaction between cardiomyocytes and macrophages. In our study, lipopolysaccharide (LPS) was used solely as an *in vitro* tool to simulate the inflammatory environment experienced by cardiomyocytes during infection or injury. The purpose was to dissect the molecular composition and function of C-lEVs secreted under defined stress conditions. Our findings revealed that lEVs derived from LPS-preconditioned cardiomyocytes (C-lEV_LPS_) regulated macrophage polarization by altering M1/M2 macrophage marker expression and suppressing inflammatory cytokine production. Further investigation demonstrated that C-lEV_LPS_ influenced macrophage polarization by interacting with the p38 MAPK pathway. Proteomic analysis showed that C-lEV_LPS_ delivers PP2AA to macrophages, thereby modulating the p38 MAPK pathway. Essentially, this research specifically looks into the involvement of C-lEVs in myocarditis, providing new insights into its pathogenesis and potential therapeutic approaches.

## 2 Materials and methods

### 2.1 Cell culture

The cardiomyocyte cell line H9C2, murine macrophage cell line RAW264.7, and human laryngeal squamous cell carcinoma Hep2 cells were purchased from Yanyi Biotech Co., Ltd. (Shandong, China). Cells were maintained in high-glucose DMEM supplemented with 10% fetal bovine serum and 1% penicillin-streptomycin (Solarbio, Shanghai, China) under conditions of 37 °C and 5% CO_2_. H9C2 cells were starved for 12 hours and then exposed to 1.5 μg/mL LPS for 24 hours, while RAW264.7 cells received a 12-hour treatment with 1 μg/mL LPS to stimulate polarization. Following this, the therapeutic impact of C-lEVs was examined by introducing 1 × 10^7^ C-lEVs particles to the culture medium. 100 nM Anisomycin(AM, MCE, #22862-76-6) was introduced into the culture medium to stimulate the MAPK signaling pathway.

### 2.2 Isolation and characterization of C-lEVs

The culture medium of H9C2 cardiomyocytes treated with PBS or LPS was collected, and the C-lEVs were obtained by differential centrifugation as previous described (9). Briefly, the cell culture medium was collected and centrifuged at 300× g at 4 °C for 10 minutes, followed by additional centrifugation steps at 2,000× g and 10,000× g at 4 °C for 20 and 30 minutes, respectively. Following resuspension with PBS, the pelleted C-lEVs were washed by recentrifugation at 10,000× g for 30 minutes at 4 °C.

Transmission electron microscopy (TEM) analyses were performed by the 120 kV transmission electron microscope (Hitachi HT-7800, Tokyo, Japan). In brief, 10 μL C-lEVs were loaded on a copper grid placed on ice for 1 minute. 10 μL of phosphotungstic acid was applied to the copper grid for 1 minute for negative staining. The grid was air-dried for several minutes before TEM imaging at 120 kV.

The size and concentration of C-lEVs were analyzed using nanoparticle tracking analysis (NS300, Malvern, UK). Following the manufacturer’s instructions, the NTA instrument was set up. The C-lEV samples were diluted to 1 mL with PBS and analyzed at room temperature.

### 2.3 RT-qPCR

RNA was isolated from cells or heart tissue by adding Trizol Reagent (AG21101, Accurate Biology), then separated by adding chloroform and centrifugation. With a reverse transcription kit Evo M-MLV RT (AG11707, Accurate Biology), 1 μg total RNA was converted into cDNA. RT-qPCR reactions were performed on a ROCHE 480II using the SYBR^®^ Green qPCR Kit (AG11701, Accurate Biology) and appropriate primers. Primer sequences are provided in **Supplementary Table S1**.

### 2.4 Western blot analysis

The cell and C-lEV proteins were obtained by adding RIPA lysis buffer supplemented with protease and phosphatase inhibitors (Beyotime Biotechnology, CN). Proteins were quantified by a BCA assay kit. After adding loading buffer, the sample was heated to 95 °C for 10 minutes to induce denaturation. Identical protein amounts were resolved using SDS-PAGE and then transferred to PVDF membranes (Millipore, USA). The membrane was blocked with 5% non-fat milk for 1–2 hours at room temperature, followed by overnight incubation at 4 °C with specific antibodies on a skaker. Membranes were then incubated with HRP-conjugated secondary antibodies, and immunoblots were visualized using Pierce ECL reagent (Thermo Fisher) through chemiluminescence. Antibodies used included Calnexin (A15631, ABclonal, 1:1000), TSG101 (ab125011, Abcam, 1:2000), β-actin (#sc47778, Santa Cruz Biotechnology, 1:3000), CD63 (ab217345, Abcam, 1:1000), Tubulin (ab6160, Abcam, 1:5000), p-p38 (4511T, Cell Signaling Technology, 1:1000), p38 (ab170099, Abcam, 1:2000), GAPDH (10494-1-AP, Proteintech, 1:5000), and PP2A (81G5, Cell Signaling Technology, 1:1000).

### 2.5 *In vitro* cellular uptake of C-lEVs

Following the manufacturer’s instructions, C-lEVs labeled with Dil fluorochrome (C1991S, Byotime) were prepared. RAW264.7 cells were stained with Actin-Tracker Green-488 (C2201S, Byotime) for 10 minutes at 37 °C and incubated with fluorescently labeled C-lEVs for 30 minutes. Cells were fixed with 4% paraformaldehyde and stained with DAPI (Sigma-Aldrich, #D9542). Using an EVOS M7000 fluorescence microscope (Invitrogen, Massachusetts, USA), images were observed.

### 2.6 Flow cytometry

RAW264.7 cells were collected and counted using an automated cell counter (CountessTM3, Thermo Fisher). 10^6^ cells were blocked with 1% bovine serum albumin (BSA, Gibco) to prevent non-specific binding, followed by incubating at 4 °C for 30 minutes with PE-CD86 monoclonal antibody (1:100; 561963, BD Biosciences). To stain with the CD206 antibody, cells were fixed and permeabilized with the intracellular staining kit (00-5523-00, eBioscience) after blocking Fc receptors, as the epitope is located in the medial segment of the cell membrane. Subsequently, PE-CD206 (1:400; 2535977, eBioscience™) staining was performed. Following staining, the cells were rinsed twice with 1% BSA and examined using a flow cytometer (ATTUNE NXT, Thermo Fisher).

### 2.7 Cell proliferation assay

Cell proliferation was assessed with a CCK-8 kit. Briefly, 2 × 10^4^ cells/well per 100 µL was seeded into 96-well plates. 24 hours after the plates were planted, each group of cells was subjected to fluid exchange with the treatment of PBS, LPS, and LPS+C-lEVs, respectively. Following a 10-hour treatment, each well received 10 µL of CCK8 reagent (Dojindo, Kumamoto, Japan) and was further incubated for 2 hours. Absorbance measurements at 450 nm were performed on a TECAN Spark microplate reader.

### 2.8 Detection of reactive oxygen species

The intracellular ROS level was measured with dichlorofluorescein diacetate (DCFH-DA) green dye. RAW264.7 cells subjected to various treatments were incubated at 37 °C for 45 min with 20 μM DCFH-DA (Beyotime Biotechnology, China; #S0033S) for staining, followed by PBS washing. Then the fluorescent intensity of cells was detected with a flow cytometer (AttuneNxT, Thermo Fisher Scientific, Massachusetts, USA) and analyzed using FlowJo 10.8.1 software.

### 2.9 Animal experiment

The Coxsackievirus B3 (CVB3, Nancy strain, USA) was amplified in Hep2 cells with titter measured via the 50% tissue culture infection dose (TCID_50_) method. The Animal Experimental Ethics Committee of Shandong Provincial Hospital approved the study’s animal experimental protocols (NSFC: 2024-178). SPF grade BALB/c male mice (18–20 g, 4 weeks old) were purchased from Beijing Vital River Laboratory Animal Technology Co., Ltd. and kept in a specific pathogen-free conditions. After a 3-day adaptive feeding period, mice were randomly assigned to one of three groups: the normal control group (NC), the CVB3 group, or the CVB3+C-lEV_LPS_ group. A dose of 200 µL 10^4^ TCID_50_ CVB3 was given to mice by intraperitoneal injection to set up viral myocarditis model, whereas the NC group received 200 µL PBS administration intraperitoneally. To investigate the effect of C-lEV_LPS_, a dose of 150 µL C-lEV_LPS_ (1 × 10^8^ particles/mL) was administered via the tail vein day 1 and day 3 following the establishment of the viral myocarditis model. Seven days after virus or PBS injection, echocardiography was performed on the mice in each group using M-mode ultrasound (SiliconWave 60, Kolo Medical Co., Ltd., Suzhou, China) to measure the ejection fraction (EF) and fractional shortening (FS) of the left ventricle. Then all mice were anesthetized, blood samples were collected in EDTA tubes, and myocardial tissue was fixed with 4% paraformaldehyde.

### 2.10 Enzyme-linked immunosorbent assay

ELISA analysis was conducted to evaluate the level of cardiac biomarker cardiac troponin (cTnT) in mice plasma. Plasma samples were centrifuged at 1,000× g at 4 °C for 15 minutes within 30 minutes of collection, and the supernatant was collected. cTnT detection was performed using a mouse ELISA kit (ReedBiotech, RE1358M) following the manufacturer’s instructions.

### 2.11 Hematoxylin-eosin and immunohistochemistry staining

Heart samples fixed with 4% paraformaldehyde were embedded in paraffin and sectioned into 5 µm white pieces. After staining with hematoxylin-eosin, samples were observed under an optical microscope. Immunohistochemistry staining was performed as previously described (35), the paraffin-embedded heart tissue was cut into 5 µm thick sections, dewaxed and rehydrated, and after antigen repair, the endogenous catalase was blocked. The slide was sealed with 1% BSA for 30 minutes, incubated with CD86 primary antibody overnight, washed, treated with a biotin-coupled secondary antibody for 30 minutes, and observed under a microscope following DAB staining. The CD206 antibody, targeting an epitope in the medial cell membrane segment, was incubated with 0.2% Triton X-100 following BSA blocking to permeabilize the membrane for antibody staining for 15 minutes. Myocardial lesion severity was scored as follows: 0 = no inflammation; 1 = 1–5 inflammatory foci (≤5% area); 2 = >5 foci or >5-20% area involvement; 3 = diffuse inflammation (>20% area), no necrosis; 4 = diffuse inflammation with necrosis.

### 2.12 PolyA-Selected mRNA-Seq

RNA was isolated by adding chloroform followed by centrifugation. Quality assessment involved agarose gel electrophoresis, Nanodrop spectrophotometry, and Qubit fluorometry. Library preparation was streamlined using a kit that combined double-strand synthesis, end repair, and dA-tailing. mRNA was enriched with VAHTS mRNA Capture Beads, fragmented, and then reverse transcribed into cDNA (9, 36). The cDNA was purified, and sequencing adapters were ligated. Library quality was assessed using the Agilent Bioanalyzer and Qubit fluorometer. Sequencing was conducted on an Illumina Novaseq6000 platform in paired-end 150 mode. This protocol generated high-quality sequencing data suited for subsequent bioinformatic analysis.

Raw data was processed with fastp (version 2.0), which removed adapter sequences, trimmed low-quality bases (quality score < 20), and filtered out reads containing more than 10% ambiguous nucleotides (N). The resulting data was referred to as clean data. These clean reads were mapped to the reference genome with HISAT2 (version 2.1.0), and the alignment quality was evaluated with RSeQC (version 3.0.1). Gene expression levels were quantified with StringTie, and differentially expressed genes were identified through DESeq2 analysis (9).

### 2.13 NanoLC-MS/MS analysis

Peptides (200 ng) were analyzed using a nano-UPLC (nanoElute2) coupled with a timsTOF Pro2 mass spectrometer (Bruker) equipped with a nano-electrospray ion source. A PePSep C18 reversed-phase column (1.9 µm, 75 µm × 15 cm, Bruker, Germany) was used for the separation. The mobile phases were H_2_O with 0.1% FA (phase A) and ACN with 0.1% FA (phase B). The sample was separated using a 60-minute gradient at a flow rate of 300 nL/min. Gradient B: 2% for 0 minutes, increasing to 22% over 45 minutes, then to 37% over 5 minutes, further to 80% over another 5 minutes, and maintained at 80% for 5 minutes (37).

Data acquisition is performed in DDA PaSEF mode, with an MS1 scanning range between 100 and 1700 m/z. In PASEF MS/MS scanning, the impact energy rises linearly with ion mobility, starting at 20 eV for 1/K0 = 0.6 Vs/cm^2^ and reaching 59 eV for 1/K0 = 1.6 Vs/cm^2^.

### 2.14 Protein identification and quantification

The vendor’s raw MS files were analyzed with SpectroMine software (v4.2.230428.52329) with the integrated Pulsar search engine. MS spectra were searched against a species-specific UniProt FASTA database (Rattus.fasta) with fixed carbamidomethylation and variable oxidation and N-terminal acetylation. Trypsin was utilized as a protease. A maximum of 2 missed cleavages was permitted. Both PSM and peptide levels were set at 0.01 false discovery rate. Peptide identification was conducted with an initial precursor and fragment mass deviation of up to 20 ppm. All other parameters were kept as default.

### 2.15 Statistical analysis

The data were statistically analyzed and plotted by GraphPad Prism9 software. *p*-values were calculated using Student’s *t*-test or one-way ANOVA. *p* < 0.05 was deemed statistically significant. All experiments were conducted a minimum of three times.

## 3 Results

### 3.1 Characterization of C-lEVs

**Figure 1:**
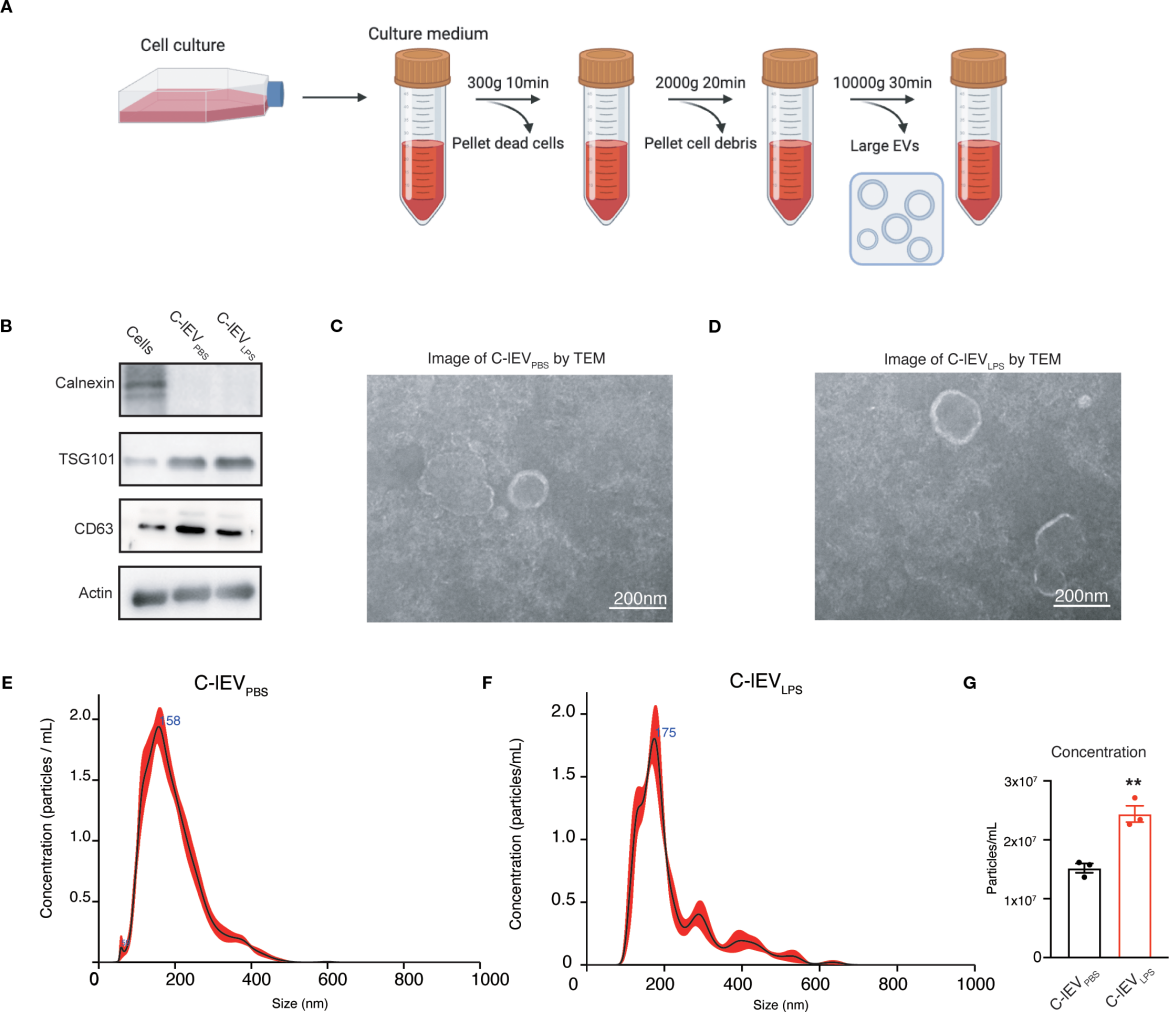
Isolation and characterization of C-lEVs. **(A)** Workflow schematic for the isolation of C-lEVs. **(B)** Western blot analysis of Calnexin and C-lEVs markers of TSG101, CD63, and Actin. **(C, D)** Representative images of C-lEV_PBS_ and C-lEV_LPS_ by TEM, scale bars: 200 nm. **(E, F)** Size distribution of C-lEV_PBS_ and C-lEV_LPS_. **(G)** Particle concentration of C-lEV_PBS_ and C-lEV_LPS_. **p < 0.01 vs. C-lEV_PBS_ group.

The rat cardiomyocyte cell line H9C2 was used for the isolation of C-lEVs. Following LPS treatment, there was a significant increase in inflammatory cytokines such as IL-6, IL-1β, and TNF-α, accompanied by a decrease in cell viability (**Supplementary Figure S1**). Differential centrifugation was used to isolate C-lEV_PBS_ and C-lEV_LPS_ from the culture medium (**Figure 1A**). Western blot analysis confirmed the presence of C-lEV markers TSG101, CD63, and Actin, while Calnexin was absent (**Figure 1B**). TEM revealed the typical membranous cup-shaped morphology of both C-lEV_PBS_ and C-lEV_LPS_ (**Figures 1C, D**). NTA-determined particle sizes predominantly ranged from 100 to 500 nm, with peak sizes of 158 nm for C-lEV_PBS_ and 171.6 nm for C-lEV_LPS_ (**Figures 1E, F**). Additionally, the particle numbers of C-lEV_LPS_ per milliliter of culture medium were significantly elevated compared to C-lEV_PBS_ (**Figure 1G**).

### 3.2 *In vitro* studies demonstrate the anti-inflammatory properties of C-lEV_LPS_

**Figure 2:**
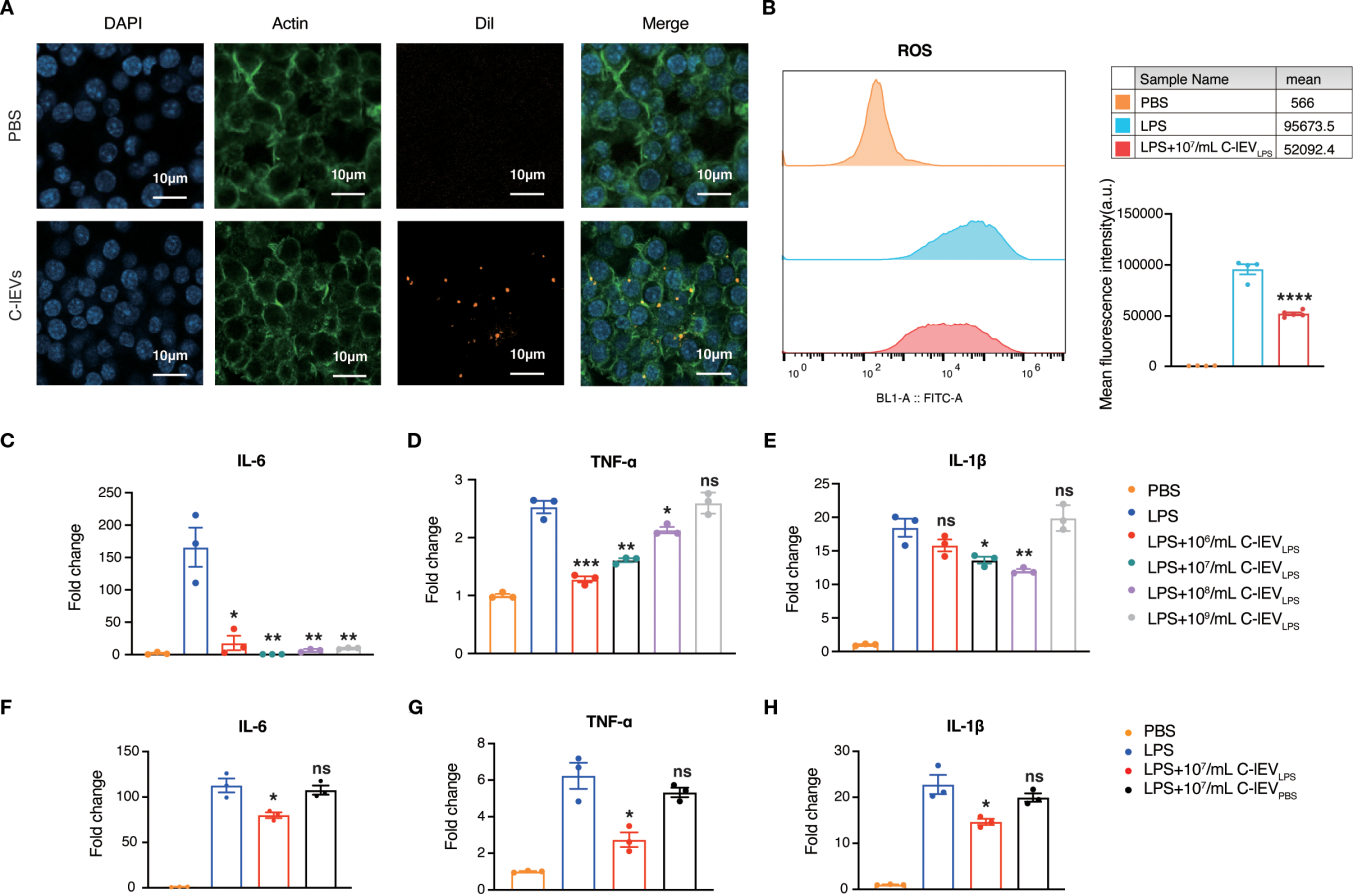
C-lEV_LPS_ suppressed LPS-stimulated inflammation in RAW 264.7 cells. **(A)** Representative images of RAW 264.7 cells internalizing C-lEV_LPS_, scale bar: 10 µm. **(B)** ROS production in RAW264.7 cells. **(C-H)** The expression levels of IL-6, TNF-α, and IL-1β in RAW264.7 cells determined by RT-qPCR under different treatments. *p < 0.05, **p < 0.01, ***p < 0.001, ****p < 0.0001, ns, nonsignificant difference vs. PBS group.

The *in vitro* effects of C-lEV_LPS_ were examined with the murine macrophage cell line RAW264.7. The internalization of C-lEV_LPS_ by RAW264.7 cells was evaluated first. Dil fluorescent dye-labeled C-lEV_LPS_ were incubated with RAW264.7 cells for 6 hours. As depicted in **Figure 2A**, C-lEV_LPS_ could be taken up by RAW264.7 cells. Emerging evidence indicated that activated macrophages can release ROS and pro-inflammatory cytokines such as TNF-α, IL-1β, and IL-6 (38). Herein, RAW264.7 cells were treated with LPS and subsequently exposed to varying concentrations of C-lEV_PBS_ or C-lEV_LPS_. Flow cytometry results indicated that ROS levels were significantly elevated upon treatment with LPS, while C-lEV_LPS_ reversed the LPS-induced increase in ROS (*p*=0.000034, **Figure 2B**). The expression levels of inflammatory cytokines TNF-α, IL-6, and IL-1β were significantly elevated in LPS-induced RAW264.7 cells (**Figures 2C-E**). Different C-lEV_LPS_ concentrations resulted in varying decreases in inflammatory cytokine expression. Notably, the 10^7^ particles/mL C-lEV_LPS_ group exerted the most potent effect, significantly suppressing the expression of all inflammatory cytokines tested (**Figures 2C-E**). Additionally, increased cell proliferation was observed following LPS stimulation, which was suppressed by C-lEV_LPS_ treatment (**Supplementary Figure S2**). In contrast, different concentrations of C-lEV_PBS_ did not influence the expression of inflammatory cytokines (**Supplementary Figure S3**). Taken together, C-lEV_LPS_ decreased the expression of inflammatory cytokines compared to C-lEV_PBS_ (**Figures 2F-H**). These data indicate that C-lEV_LPS_ treatment exerts anti-inflammatory effects on macrophages.

### 3.3 C-lEV_LPS_ alleviated CVB3-induced myocarditis in mice

**Figure 3:**
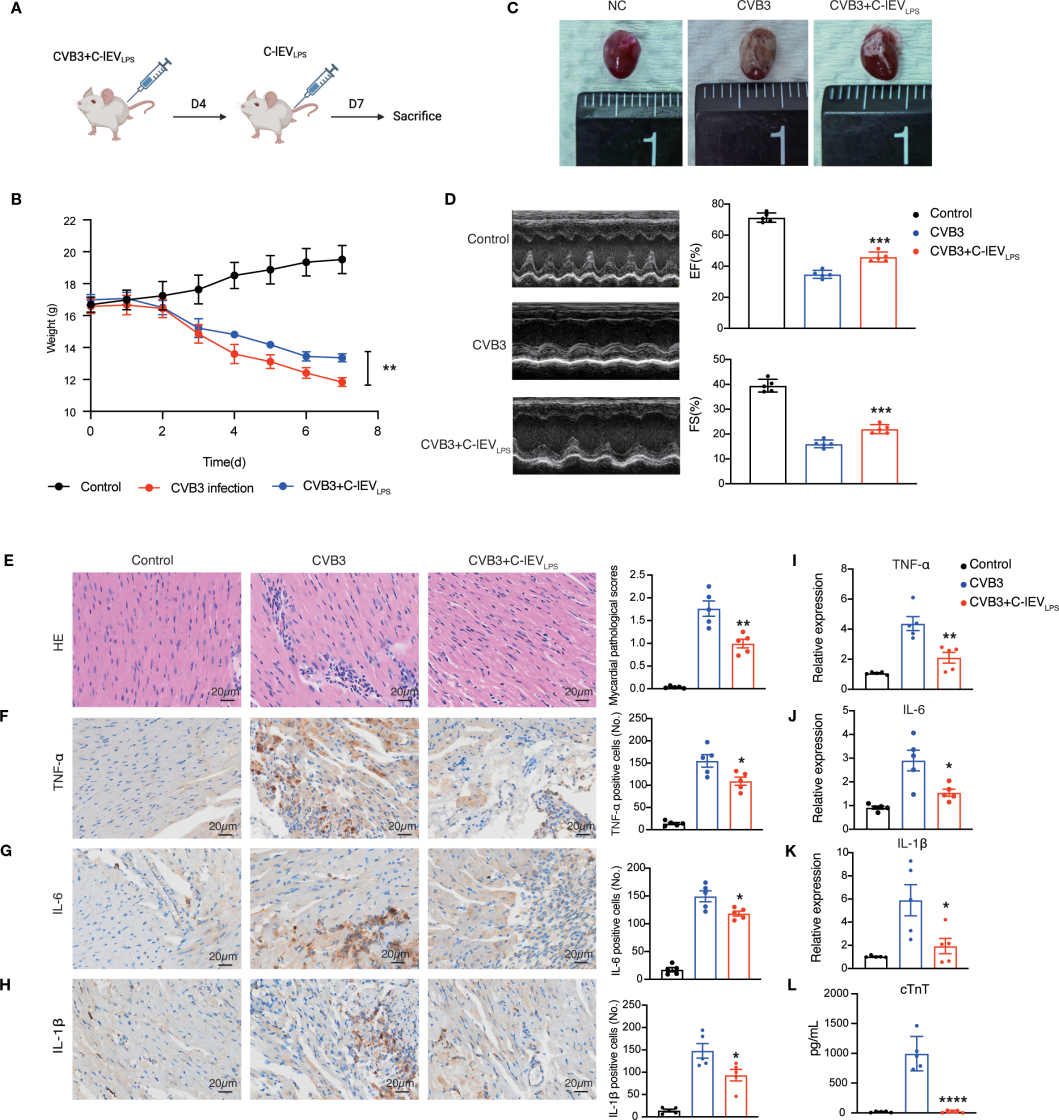
C-lEV_LPS_ treatment relieved CVB3-induced myocarditis *in vivo*. **(A)** Animal treatment illustration. **(B)** Appearance of the heart after different treatments. **(C)** Changes in mice body weight among different treatment groups. **(D)** Left panel, representative echocardiographic images of the mice. Right panel, statistical analysis results of left ventricular EF and FS. **(E)** HE staining images and pathological scores of myocardial tissues, scale bar: 20 µm. **(F-H)** Analysis of immunohistochemical staining for inflammatory cytokines expression and semiquantitative histological analysis, scale bar: 20 µm. **(I-K)** The expression levels of TNF-α, IL-6, and IL-1β in mice myocardial tissue. **(L)** The serum levels of cTnT in mice. (n = 5) *p < 0.05, **p < 0.01, ***p < 0.001, ****p < 0.0001 vs. CVB3 group.

To evaluate the role of C-lEV_LPS_ in myocarditis, BALB/c mice were injected with CVB3 to induce viral myocarditis. On days 1 and 4 post-CVB3 injection, the mice were administered 150 μL of C-lEV_LPS_ (10^8^ particles/mL) or PBS (carrier solution) via tail vein injection (**Figure 3A**). As expected, C-lEV_LPS_ accumulated in the mouse hearts after tail vein injection (**Supplementary Figure S4**). On day 7, C-lEV_LPS_ mildly reversed the weight loss observed after CVB3 infection (**Figure 3B**). Thus, cardiac function was assessed by echocardiography. As expected, mice induced by CVB3 exhibited cardiac dysfunction, as evidenced by decreased ejection fraction (EF) (*p*=0.0003) and fractional shortening (FS) (*p*=0.0005). Strikingly, the cardiac dysfunction was remarkably attenuated upon treatment with C-lEV_LPS_ (**Figure 3C**). However, there were no significant differences in left ventricular posterior wall thickness (LVPW) or left ventricular mass (LV mass Cor) between the CVB3 group and the C-lEV_LPS_ group (**Supplementary Figure S5**). In addition, Kaplan–Meier survival analysis showed no statistically significant differences in survival rates among these groups during the observation period (**Supplementary Figure S5**).

Then the myocardial tissue was collected. The myocardial surfaces of mice in the NC group appeared smooth, while mice with viral myocarditis showed patch-like lesions in the heart. Notably, upon treatment with C-lEV_LPS_, the area of patch-like lesions was significantly reduced in the heart (**Figure 3D**). H&E staining indicated significantly higher pathology scores in the CVB3 group of mice compared to the NC group, while the CVB3 + C-lEV_LPS_ group exhibited notably reduced scores relative to the CVB3 group (**Figure 3E**). IHC staining and RT-qPCR analysis showed that TNF-α, IL-6, and IL-1β expressions were significantly elevated in the hearts of the mice in the CVB3 group compared to the NC group. Conversely, these cytokine expressions were reduced significantly in the CVB3 + C-lEV_LPS_ group compared to the CVB3 group (**Figures 3F-K**). Additionally, ELISA analysis revealed that plasma cTnT levels were significantly elevated in CVB3-infected mice and markedly decreased in the CVB3 with C-lEV_LPS_ group (**Figure 3L**). Collectively, these results indicate that C-lEV_LPS_ treatment reduces the inflammatory response in myocarditis.

### 3.4 C-lEV_LPS_ induces macrophage polarization from M1 toward M2

**Figure 4:**
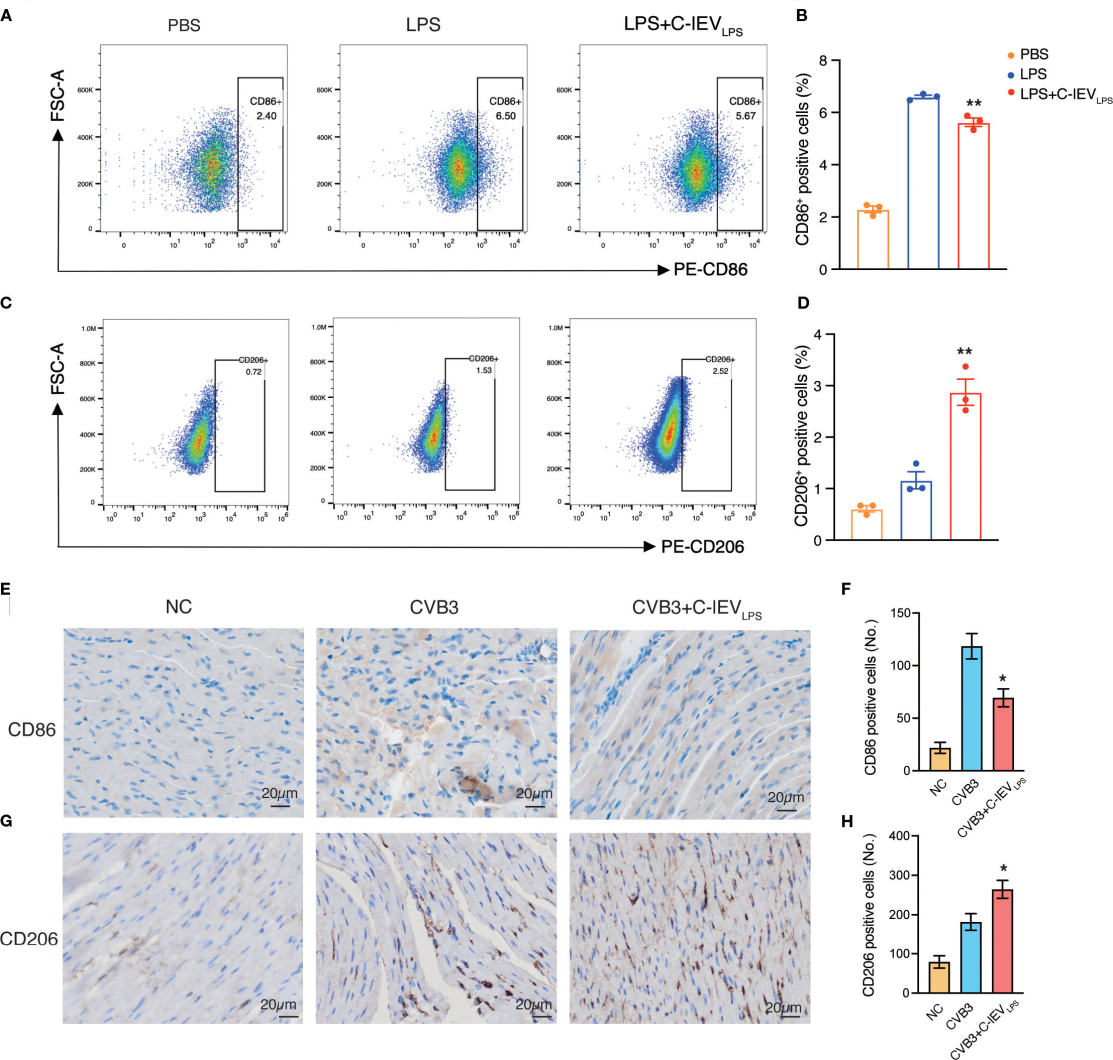
C-lEV_LPS_ treatment regulated macrophage polarization both *in vitro* and *in vivo*. **(A, B)** Flow cytometry analysis of CD86 positive RAW 264.7 cells and quantitative data analysis. **(C, D)** Flow cytometry analysis of CD206 positive RAW 264.7 cells and quantitative data analysis. **(E, F)** Analysis of immunohistochemical staining for CD86 and semiquantitative histological analysis (n=5), scale bar: 20 µm. **(G, H)** Analysis of immunohistochemical staining for CD206 and semiquantitative histological analysis (n=5), scale bar: 20 µm. *p < 0.05, **p < 0.01 vs. LPS or CVB3 group.

Macrophages are crucial in the early development of myocarditis as the main inflammatory and infiltrating cells (5, 7). They can differentiate into pro-inflammatory M1 or anti-inflammatory M2 phenotypes based on the cardiac environment (39). Flow cytometry was used to evaluate macrophage phenotypes and determine if C-lEV_LPS_ reduces inflammation by influencing macrophage polarization. The results indicated that the proportion of CD86-positive M1 macrophages significantly increased after LPS treatment, whereas C-lEV_LPS_ treatment reduced this proportion (*p*=0.008, **Figures 4A, B**). Conversely, the proportion of CD206-positive M2 macrophages increased after LPS treatment and was further elevated with C-lEV_LPS_ treatment (*p*=0.0049, **Figures 4C, D**).

Additionally, to verify whether C-lEV_LPS_ could influence macrophage polarization *in vivo*, we performed IHC staining of CD86 and CD206 on the myocardial tissues in the mice. C-lEV_LPS_ treatment modulated macrophage infiltration, reducing the presence of M1 macrophages while enhancing the recruitment of M2 macrophages (**Figures 4E-H**). These findings suggest that C-lEV_LPS_ treatment induced macrophage polarization toward an M2-like phenotype while inhibiting M1 polarization.

### 3.5 C-lEV_LPS_ regulates macrophage polarization via the p38 MAPK pathway

**Figure 5:**
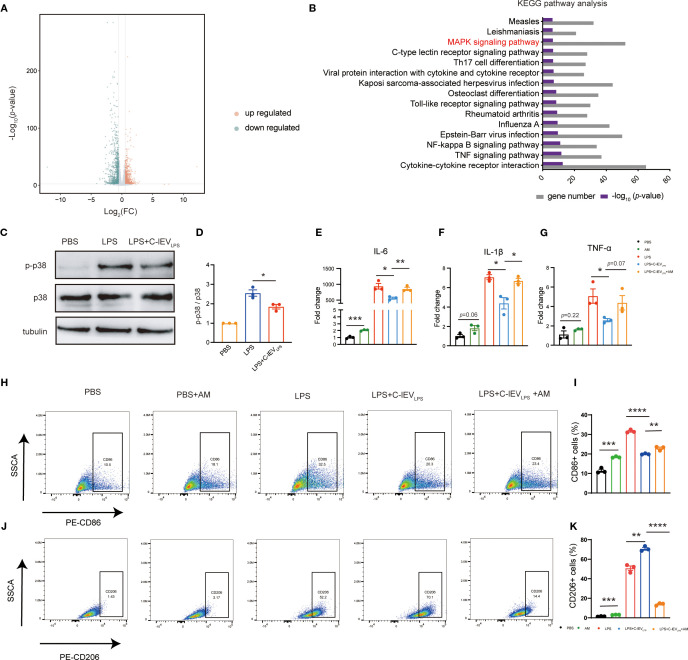
C-lEV_LPS_ regulates macrophage polarization by the p38 MAPK pathway. **(A, B)** Transcriptomic alterations in LPS-stimulated RAW 264.7 cells following treatment with C-lEV_LPS_ revealed by RNA-seq analysis. **(C, D)** Representative bands of p-p38/p38 levels by Western blot, with semiquantitative analysis, in RAW 264.7 cells subjected to various treatments. **(E-G)** Expression levels of pro-inflammatory gene profiles in RAW 264.7 cells. **(H, I)** Flow cytometry analysis of CD86-positive RAW 264.7 cells and quantitative data analysis. **(J, K)** Flow cytometry analysis of CD206-positive RAW 264.7 cells and quantitative data analysis. *p < 0.05, **p < 0.01, ***p < 0.001, ****p < 0.0001.

To elucidate the mechanism by which C-lEV_LPS_ regulates macrophage polarization, RNA sequencing was conducted to identify gene expression differences in macrophages upon treatment with or without C-lEV_LPS_. The volcano plot analysis revealed 941 upregulated genes (*p* < 0.05, log_2_FC > 0.5) and 1048 downregulated genes (*p* < 0.05, log_2_FC < −0.5) (**Figure 5A**).

Notably, gene ontology (GO) analysis of differentially expressed genes indicated significant enrichment of the MAPK pathway (**Figure 5B**). Given that the p38 MAPK pathway is involved in macrophage polarization (40, 41), we hypothesized that C-lEV_LPS_ modulates macrophage polarization through the p38 MAPK pathway. Blot analysis demonstrated that LPS increased p38 phosphorylation, an effect reversed by C-lEV_LPS_, pointing to C-lEV_LPS_-mediated inhibition of the p38 MAPK pathway (**Figures 5C, D**).

To confirm that C-lEV_LPS_ influences macrophage polarization through the p38 MAPK pathway, LPS-preconditioned RAW264.7 cells were co-cultured with C-lEV_LPS_ and the p38 MAPK agonist AM. RT-qPCR analysis demonstrated that treatment with C-lEV_LPS_ significantly reduced the levels of pro-inflammatory cytokines IL-6, IL-1β, and TNF-α compared to the LPS group. However, co-treatment with AM eliminates the anti-inflammatory effects of C-lEV_LPS_ (**Figures 5E–G**). Flow cytometry analysis showed that treatment with C-lEV_LPS_ significantly increased the proportion of CD206-positive M2 macrophages and decreased the proportion of CD86-positive M1 macrophages compared to the LPS group. Notably, the addition of AM reversed the effects of C-lEV_LPS_, which reduced the proportion of M2 macrophages and increased M1 macrophages (**Figures 5H–K**). Collectively, these findings suggest that C-lEV_LPS_ modulates macrophage polarization, at least in part, through the p38 MAPK signaling pathway.

### 3.6 PP2AA serves a potential regulator from C-lEV_LPS_ mediating macrophage polarization

**Figure 6:**
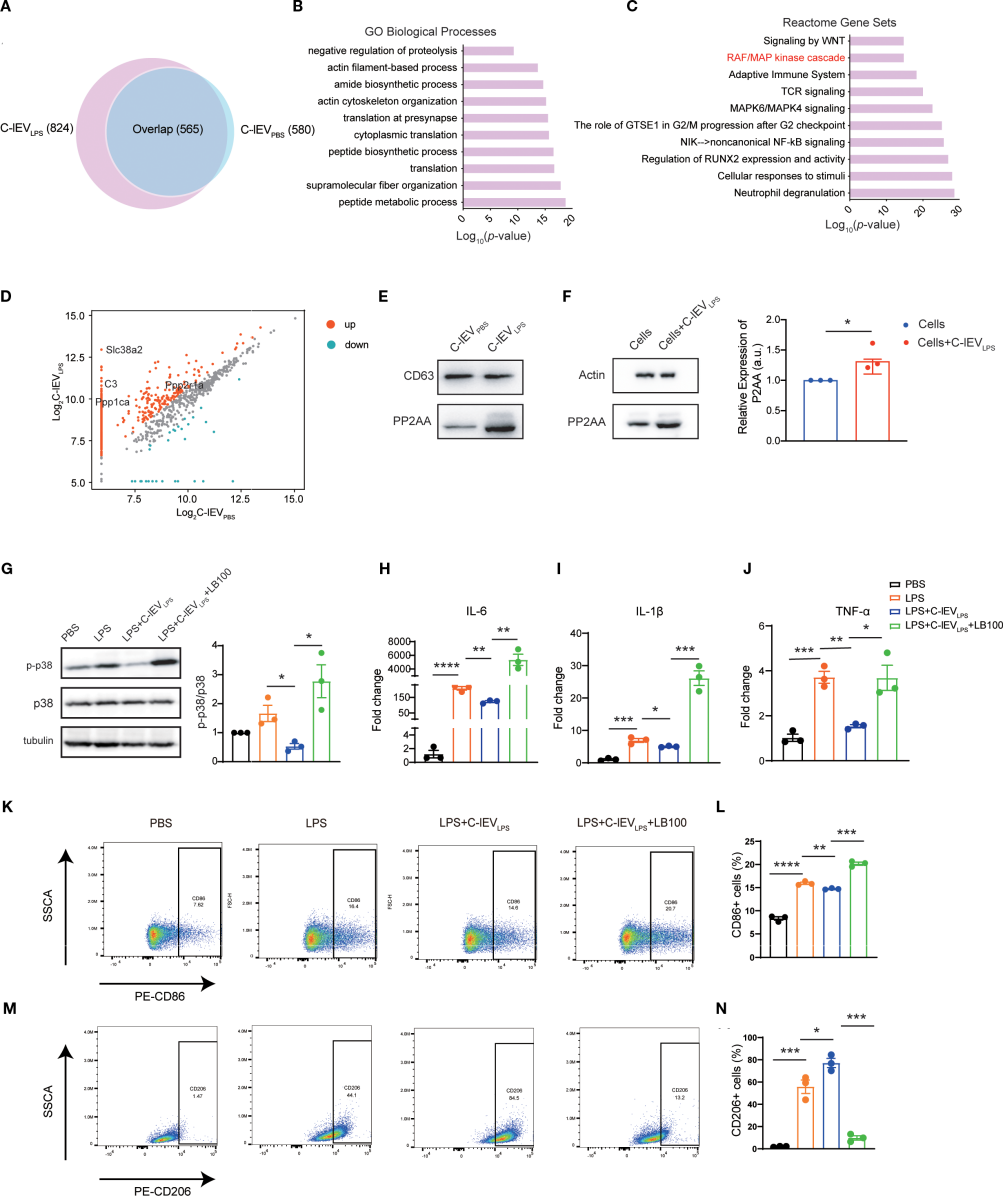
C-lEV_LPS_ encapsulates PP2AA and regulates the p38-MAPK pathway of macrophage by synthesizing PP2A. **(A)** Venn diagram depicting the common and total proteins in C-lEV_LPS_ vs. C-lEV_PBS_. **(B)** Gene ontology analysis of proteins enriched in C-lEV_LPS_ focused on biological processes. **(C)** Reactome pathway analysis conducted on proteins enriched in C-lEV_LPS_. **(D)** Volcano plot illustrating the differentially enriched proteins in C-lEV_LPS_ vs C-lEV_PBS_. **(E)** Representative bands of PP2A and CD63 in C-lEV_LPS_ and C-lEV_PBS_ by Western blot. **(F)** Western blot showing PP2A and actin expression in RAW264.7 cells. **(G)** Representative bands of p-p38/p38 levels by Western blot, with semiquantitative analysis, in RAW 264.7 cells subjected to various treatments. **(H-J)** Expression levels of pro-inflammatory gene profiles in RAW 264.7 cells. **(K, L)** Flow cytometry analysis of CD86-positive RAW 264.7 cells and quantitative data analysis. **(M, N)** Flow cytometry analysis of CD206-positive RAW 264.7 cells and quantitative data analysis. *p < 0.05, **p < 0.01, ***p < 0.001, ****p < 0.0001.

EVs are known to contain multiple proteins relevant to various biological processes (34). Based on label-free proteomics analysis, C-lEV_LPS_ and C-lEV_PBS_ exhibited distinct protein compositions, with 824 and 580 proteins identified, respectively (**Figure 6A**). Then GO analyses of biological processes and reactome gene set analysis were performed. GO biological process analysis (**Figure 6B**) revealed that the “peptide metabolic process” was enriched in C-lEV_LPS_ proteins significantly. Reactome gene set analysis revealed that these proteins are associated with several signaling pathways, particularly those involved in regulating the inflammatory response, including the “RAF/MAPK kinase cascade”, “Adaptive immune system”, “TCR signaling”, “MAPK6/MAPK4 signaling”, and “NIK-noncanonical NF-κB signaling” pathways (**Figure 6C**).

Among the proteins associated with the “RAF/MAPK kinase cascade”, Ppp2r1a was identified as upregulated in C-lEV_LPS_ (**Figure 6D**), which was validated by Western blot analysis (**Figure 6E**). Ppp2r1a encodes PP2AA, a subunit of protein phosphatase 2A (PP2A), an important serine-threonine phosphatase capable of dephosphorylating p38 MAPK. It has been reported that PP2AA can recruit other subunits of PP2A (42). Therefore, we hypothesized that the upregulated PP2AA in C-lEV_LPS_ is delivered to macrophages, where it recruits additional PP2A subunits to dephosphorylate p38 MAPK.

To confirm that C-lEV_LPS_ transports PP2AA to macrophages, RAW264.7 cells were incubated with C-lEV_LPS_ for 12 hours. Western blot showed a significant increase in PP2AA expression in RAW264.7 cells treated by C-lEV_LPS_ (**Figure 6F**). Overall, these data indicate that C-lEV_LPS_ has the capability to deliver PP2AA to macrophages, thereby promoting the formation of PP2A complexes, which may subsequently dephosphorylate p38 MAPK.

To further confirm that the effects of C-lEV_LPS_ are mediated through PP2A, LPS-preconditioned RAW264.7 cells were co-cultured with C-lEV_LPS_ and the PP2A inhibitor LB-100. Western blot analysis revealed that C-lEV_LPS_ reduced the phosphorylation level of p38 after LPS stimulation, while the use of LB-100 diminished this effect (**Figure 6G**). Meanwhile, RT-qPCR analysis revealed that treatment with C-lEV_LPS_ significantly reduced the mRNA expression of pro-inflammatory cytokines IL-6, IL-1β, and TNF-α in LPS-stimulated RAW264.7 cells compared to the LPS group. Importantly, co-treatment with LB-100 eliminates the anti-inflammatory effects of C-lEV_LPS_ (**Figures 6H–J**).

Flow cytometry results showed that treatment with C-lEV_LPS_ decreased the proportion of CD86-positive M1 macrophages (**Figures 6K, L**) and increased the proportion of CD206-positive M2 macrophages (**Figures 6M, N**) relative to the LPS group. However, the addition of LB-100 reversed these effects resulting in increased percentage of M1 macrophages and reduced percentage of M2 macrophages (**Figures 6K-N**).

Overall, these data indicate that C-lEV_LPS_ has the capability to deliver PP2AA to macrophages, which may subsequently dephosphorylate p38 MAPK and exert anti-inflammatory effects.

## 4 Discussion

Myocarditis is characterized by the infiltration of various inflammatory cells into myocardial tissue (43, 44), with macrophages being the primary immune cells (5). Viral infections are the main cause of myocarditis (45, 46), where viruses enter cardiomyocytes via virus-specific receptors, activating innate immune responses that include macrophage infiltration (47). However, the fundamental mechanisms underlying the crosstalk between cardiomyocytes and macrophages in myocarditis remain largely unexplored.

Existing literature has shown that EVs can alter the polarization state of macrophages during cardiac injury. For example, sEVs secreted by M2 macrophages promote the conversion of macrophages toward the M2 phenotype in viral myocarditis (48), while sEVs secreted by ischemic cardiomyocytes act on macrophages, modulating their inflammatory cytokine expression profile and enhancing their adhesion to fibronectin (49). However, the effects of lEVs secreted by cardiomyocytes under inflammatory conditions on macrophages remain unexplored. In contrast to previous studies, our research fills this gap by revealing how lEVs derived from inflamed cardiomyocytes influence macrophages in myocarditis.

In this study, the *in vitro* model employed LPS to stimulate inflammatory signaling, primarily activating the TLR4-NF-κB pathway (50); the *in vivo* model used CVB3 infection, which triggers cytoplasmic RIG-I-like receptors (51) and, to a lesser extent, TLR4 signaling (52). Although the upstream receptors that initiate the two models differ, the literature has reported that the capsid protein of CVB3 can crosstalk with TLR4 and induce inflammasome activation (52, 53). The choice of LPS for *in vitro* work was indeed due to CVB3 infection causing extensive cell death and abundant viral release, making it almost impossible to obtain pure, virus-free C-lEVs, whereas LPS-induced sterile inflammation can be readily washed out of C-lEV preparations.

We found that certain doses of C-lEV_LPS_ but not C-lEV_PBS_ inhibited the secretion of inflammatory cytokines in macrophages and ameliorated myocardial inflammation in mice. In addition, our findings indicate that the culture medium of LPS-induced H9C2 cells contained approximately 2–3 × 10^7^ C-lEVs per milliliter. Similarly, the most effective concentration for macrophages is 10^7^ particles per milliliter, which is comparable to the concentration secreted by cardiomyocytes. Based on our experimental results and current literature, we speculate that only C-lEV_LPS_ at near-physiological concentrations exhibits the expected anti-inflammatory effects, while excessively high doses (e.g., 10^9^/mL) may lose their regulatory capacity. This indicates that the amount of large EVs released by cardiomyocytes under inflammatory conditions is sufficient to alter macrophage polarization. Although these findings provide valuable insights, more detailed mechanistic investigation is needed to thoroughly explain this observation. Considering that the normal blood volume of a mouse is approximately 72 mL/kg, a 20 g mouse has a blood volume of approximately 1.44 mL (54). To achieve a concentration of 10^7^ particles/mL in mouse blood, we injected 150 µL of C-lEV_LPS_ at 10^8^ particles/mL, which alleviated myocarditis in mice.

**Figure 7:**
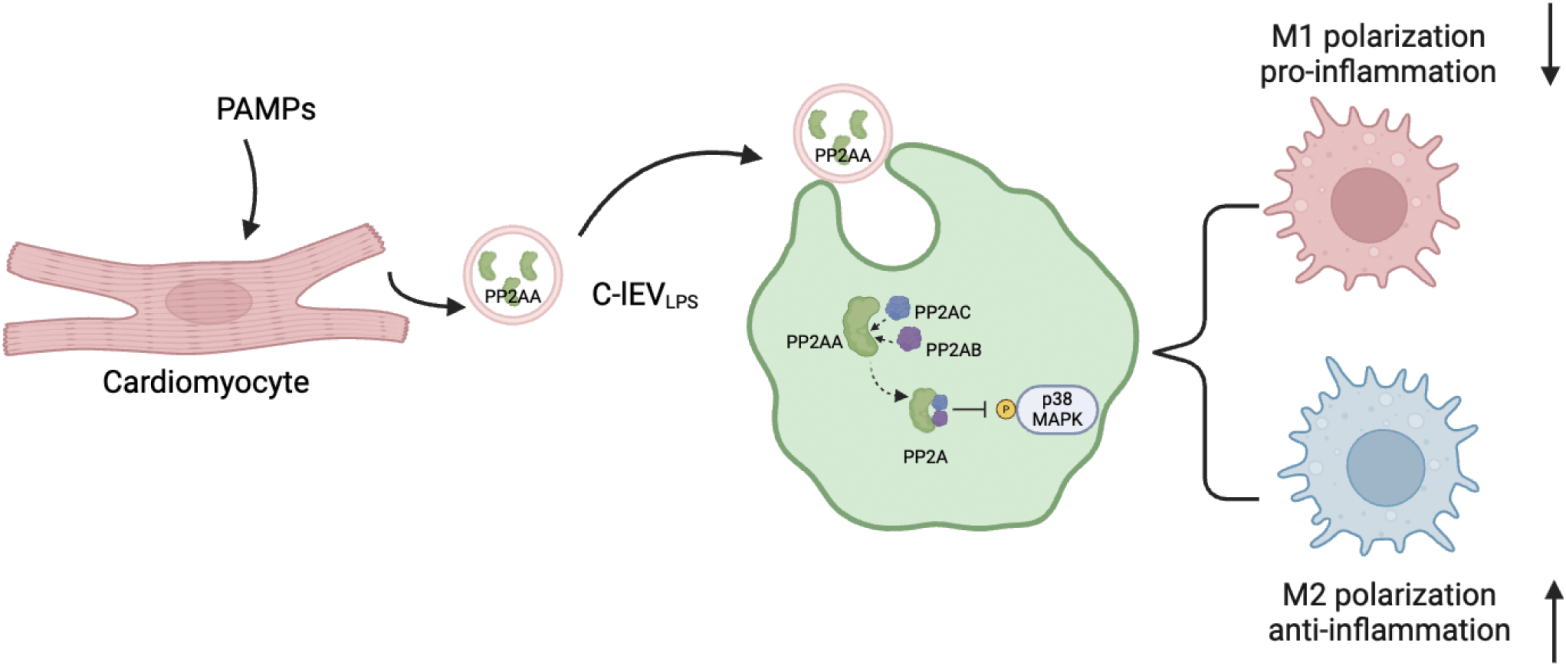
Proposed model explaining how C-lEV_LPS_ derived from inflammatory cardiomyocytes regulate macrophage polarization via MAPK pathway. The diagram is created by Biorender.

Besides, in this study we found that C-lEV_LPS_ altered the M1/M2 macrophage ratio both *in vivo* and *in vitro*. RNA sequencing suggested that the effects of C-lEV_LPS_ may be mediated through the p38 MAPK pathway, and this was further confirmed by Western blot analysis, which demonstrated that C-lEV_LPS_ could regulate the phosphorylation level of p38. Additionally, proteomic analysis identified an increase in PP2AA, a subunit of PP2A, in C-lEV_LPS_. PP2A is a crucial serine-threonine phosphatase capable of dephosphorylating p38 MAPK. PP2AA exhibits a horseshoe-shaped structure that facilitates the recruitment of other PP2A subunits (42). In addition, the PP2A inhibitor abrogated the anti-inflammatory effects of C-lEV_LPS_. These results suggest that C-lEV_LPS_ regulates the p38 MAPK pathway through PP2A (**Figure 7**). Taken together, these results suggest that C-lEV_LPS_ regulates the p38 MAPK pathway through PP2A.

Nevertheless, our study has some limitations that should be addressed in future research. First, our study uses LPS to stimulate H9C2 cardiomyocytes *in vitro* and CVB3 to induce myocarditis *in vivo*. Despite possible mechanistic differences, it is technically challenging to obtain C-lEVs completely free of infectious viral particles following CVB3 exposure. Moreover, the recent evidence indicates that CVB3 may also engage TLR4 signaling (52, 53), suggesting an overlap in the downstream pathways activated by CVB3 and LPS. Nonetheless, we recognize that viral myocarditis is more clinically relevant, and our current approach represents a compromise to balance experimental feasibility and mechanistic insight. Future studies employing advanced virus inactivation methods and refined cell models will be necessary to distinguish these mechanisms more clearly.

Second, macrophage polarization is an extremely complex process that is regulated by multiple signaling pathways (49, 55). While our data indicate that the regulatory function of C-lEV_LPS_ is mediated through the MAPK signaling pathway, we acknowledge the potential involvement of additional pathways and do not discount their significance. RNA sequencing revealed that C-lEV_LPS_ modulates numerous pathways, including the NF-κB signaling pathway, which may influence macrophage polarization. Therefore, the immune regulatory mechanisms of C-lEV_LPS_ require further investigation.

Third, we did not assess the long-term effects of C-lEV_LPS_ on myocarditis progression. Myocarditis is a dynamic disease that progresses through three distinct phases: the acute phase (characterized by viral infection and initial inflammatory response), the subacute phase (involving immune-mediated myocardial injury), and the chronic phase (which may lead to fibrosis and dilated cardiomyopathy). Our study focused solely on the acute phase, leaving open the question of whether C-lEV_LPS_ administration could influence later stages, particularly fibrosis and cardiac remodeling. Future studies should evaluate the impact of C-lEV_LPS_ at different disease stages to determine its full therapeutic potential.

Currently, there is no specific therapy for myocarditis, and symptomatic management remains the primary method. Research indicates that lncRNA AK083884 from M2 macrophage exosomes safeguards mice against CVB3-induced viral myocarditis (48). Besides, a recent study suggests that large EVs contain higher levels of cargo molecules, including proteins and RNAs, and have segregated plasma membrane domains compared to small EVs (34). This enhances their likelihood of fusing with target cells. In a study about myocardial infarction, cardiomyocyte-derived large EVs, but not small EVs, modulate the release of CCL2, CCL7, and IL-6 from cardiac monocytes (56). Leveraging a controllable in vitro LPS-induced inflammatory model, we found that inflammation-induced C-lEVs can modulate macrophage polarization and attenuate inflammation both in vitro and in vivo. This suggests that specific cargo proteins within C-lEVs (e.g., PP2A-A) could be extracted and employed as novel intercellular signaling vehicles, providing a potential therapeutic avenue for the treatment of myocarditis.

## Data Availability

The datasets presented in this study can be found in online repositories. The names of the repository/repositories and accession number(s) can be found below: https://www.ncbi.nlm.nih.gov/geo/, GSE279575 http://www.proteomexchange.org/, PXD057460.
