# Supplementary material for: Large extracellular vesicles derived from LPS-preconditioned cardiomyocytes alleviate myocarditis via mediating macrophage polarization and modulating p38 MAPK pathway
Source: Front Immunol. 2025 Sep 9;16:1629676. doi: 10.3389/fimmu.2025.1629676 (PMC12454077; doi:10.3389/fimmu.2025.1629676)
Supplement: Supplementary file 1 [file DataSheet1.docx]

Supplementary Material

# Supplementary Figures and Tables

## Supplementary Figures

**
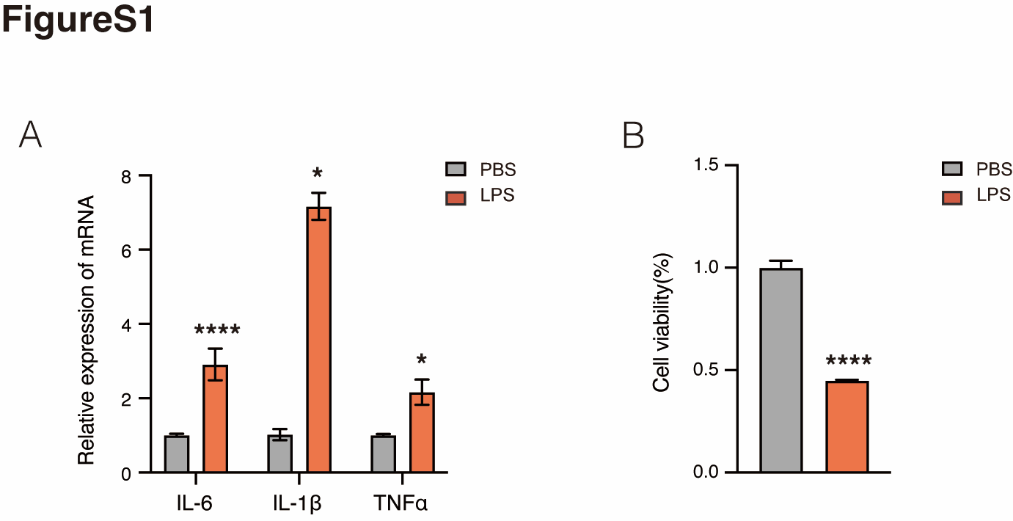
**

**Supplementary Figure 1.** (A) Expression of inflammatory cytokines in LPS-stimulated H9C2 cells. (B) H9C2 cell viability under various treatments.

**
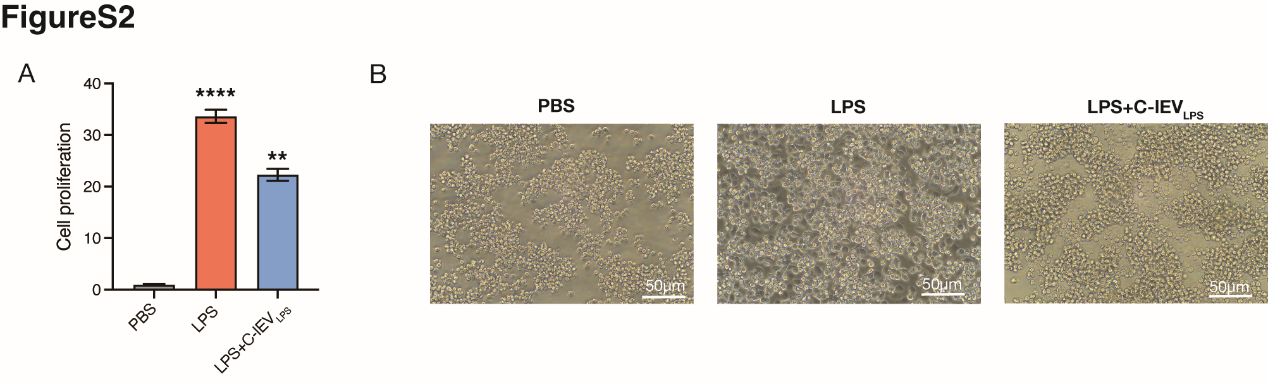
**

**Supplementary Figure 2.** (A) Raw264.7 cells proliferation under different treatments. (B) Raw264.7 cells morphology under different treatments.

**
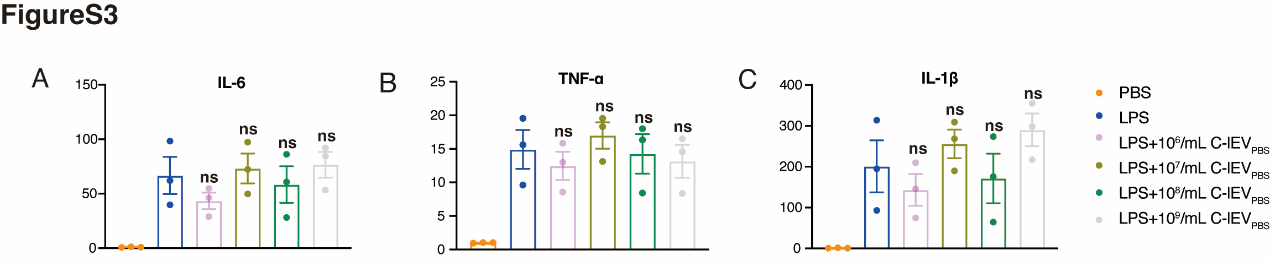
**

**Supplementary Figure 3.** The expression levels of IL-6, TNF-α, and IL-1β in RAW264.7 cells determined by RT-qPCR under different treatments. ns, nonsignificant difference vs. PBS group.


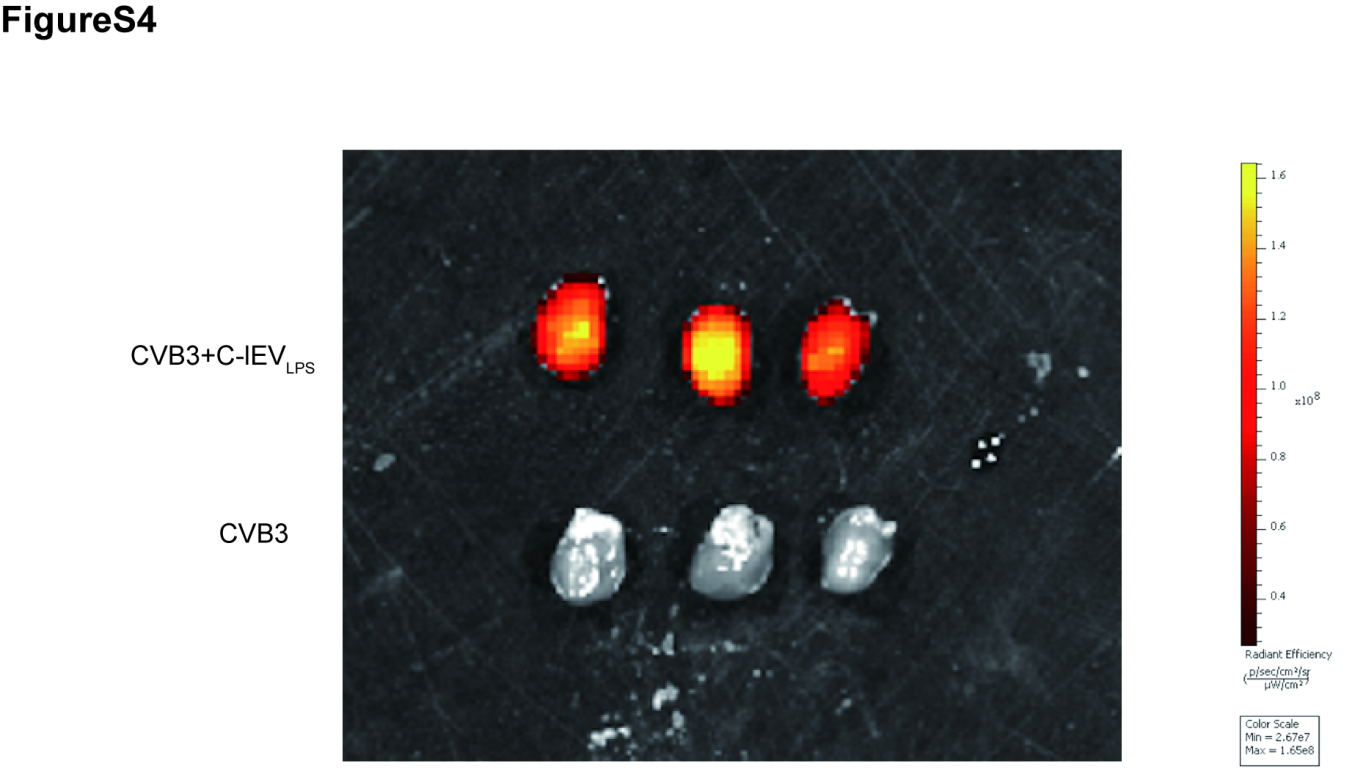


**Supplementary Figure 4.** Representative IVIS images of mice hearts after administration of vehicle and C-lEV_LPS_.


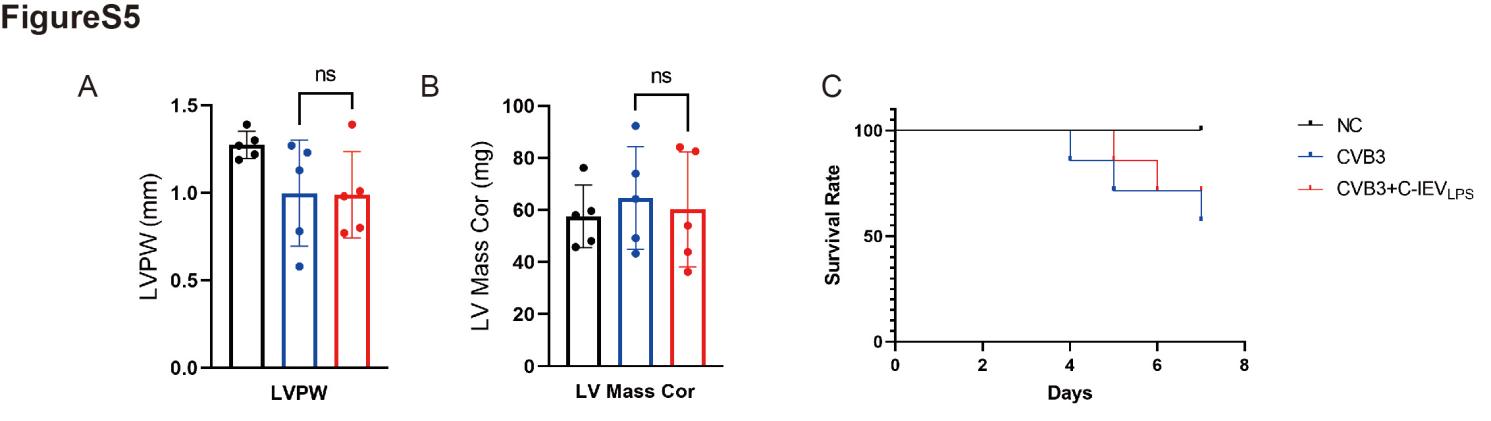


**Supplementary Figure 5.**  (A-B) Statistical analysis results of LVPW and LV Mass Cor. (C) Kaplan-Meier survival analysis of mice in different group.

**1.2 Supplementary Table 1**

The primer sequences used in qPCR detection

| Gene | Primer |
| --- | --- |
| Musculus-IL6 | F: 5’ CTTCTTGGGACTGATGCTGGT 3’  R: 5’ CTCTGTGAAGTCTCCTCTCCG 3’ |
| Musculus-TNFα | F: 5’ AGCCGATGGGTTGTACCTTG 3’  R: 5’ ATAGCAAATCGGCTGACGGT 3’ |
| Musculus-IL1β | F: 5’ GTGTCTTTCCCGTGGACCTT 3’  R: 5’ AATGGGAACGTCACACACCA 3’ |
| Musculus-CD206 | F: 5’ GCTTCCGTCACCCTGTATGC 3’  R: 5’ CTGCTCCACAATCCCGAACC 3’ |
| Musculus-TGFβ | F: 5’ AGCTGCGCTTGCAGAGATTA 3’  R: 5’ AGCCCTGTATTCCGTCTCCT 3’ |
| Musculus-IL10 | F: 5’ CACTACCAAAGCCACAAGGCA 3’  R: 5’ GAGCAGGCAGCATAGCAGTG 3’ |
| Musculus-ACTIN | F: 5’ GGCTGTATTCCCCTCCATCG 3’  R: 5’ CCAGTTGGTAACAATGCCATGT 3’ |
